# A new late Neanderthal from Crimea reveals long-distance connections across Eurasia

**DOI:** 10.1073/pnas.2518974122

**Published:** 2025-10-27

**Authors:** Emily M. Pigott, Konstantina Cheshmedzhieva, Elke Zeller, Laura G. van der Sluis, Manasij Pal Chowdhury, Maddalena Gianni, Emese Végh, Thorsten Uthmeier, Victor Chabai, Marylène Patou-Mathis, Petra G. Šimková, Jana N. Voglmayr, Gerhard W. Weber, Ron Pinhasi, Axel Timmermann, Martin Kuhlwilm, Katerina Douka, Thomas Higham

**Affiliations:** ^a^Department of Evolutionary Anthropology, University of Vienna, Vienna 1030, Austria; ^b^Human Evolution and Archaeological Science Network, Vienna 1030, Austria; ^c^Department of Geosciences, University of Arizona, Tuscon, AZ 85721; ^d^Institut für Ur- und Frühgeschichte, Friedrich-Alexander-Universität (FAU) Erlangen-Nürnberg, Erlangen D-91054, Germany; ^e^Institute of Archaeology, National Ukrainian Academy of Science, Kyiv 04210, Ukraine; ^f^Muséum National d’Histoire Naturelle, Institut de Paléontologie Humaine, Paris 75013, France; ^g^Institute for Basic Science (IBS) Center for Climate Physics, Busan 46241, South Korea; ^h^Department of Integrated Climate System Science, Pusan National University, Busan 46241, South Korea

**Keywords:** Paleolithic archaeology, Neanderthals, paleoproteomics, radiocarbon dating, ancient DNA

## Abstract

The Crimean Peninsula contains several important Middle and Upper Paleolithic sites, including Starosele, Kabazi II, and Siuren I. The region has been considered a potential refugium for Neanderthals before their replacement by *Homo sapiens*. However, no genetic data have been obtained from any of these late Neanderthals, some being inaccessible or badly preserved. Starosele is a notable site which has undergone excavations in recent years. We used collagen peptide mass fingerprinting (Zooarchaeology by Mass Spectrometry, ZooMS), to screen for potential human remains among thousands of fragmented bones from the site. Of the 150 bone fragments we analyzed, 97.3% had sufficient collagen preservation for taxonomic identification. Our results suggest Paleolithic humans primarily hunted horses. One ~5 cm bone fragment yielded peptide mass fingerprints matching *Hominidae*. Radiocarbon dating revealed an age range of 46 to 45,000 y old, close to the transition from the disappearance of Neanderthals to the dispersal of *H. sapiens* in western Europe. We sequenced a twofold coverage mitochondrial genome from this bone, indicating the individual belongs to the Neanderthal lineage. The mitogenome clusters with other Neanderthal mitogenomes previously generated from the Russian Altai region. Alongside this, an analysis of the lithic corpus from both regions suggests that a wider Neanderthal dispersal, linked to the Micoquian stone tool industry, occurred after ~60,000 y ago. We assessed the paleoclimate connection (temperature and precipitation) between these locations and identified a high habitat suitability corridor along 55°N, suggesting that the long-distance movement of Neanderthals would have been facilitated by periods of favorable climate.

## Significance

The Crimean Peninsula contains key Middle to Upper Paleolithic transitional archaeological sites, including the site of Starosele, where we identified a new Neanderthal; Star 1. This study highlights the integration of Zooarchaeology by Mass Spectrometry (ZooMS), radiocarbon dating, and ancient DNA analysis to uncover rare hominin remains and enhance our understanding of late Neanderthal populations in this region. Genetically, Star 1 is closely related to Neanderthals from the Altai via its mitochondrial DNA, suggesting long-range migrations of Neanderthal groups across Eurasia. These migrations during favorable climatic conditions likely involved the spread of the Micoquian lithic tradition, indicating both cultural continuity and regional mobility during the Late Pleistocene.

The Middle to Upper Paleolithic transition marks the biocultural transformation from a Neanderthal-dominated western Eurasia to one exclusively populated by *Homo sapiens*. Documenting the precise nature and timing of this transition requires a careful analysis of the excavated remains from the numerous archaeological sites in the wider region, along with the application of the latest biomolecular and chronometric methods. Precise radiocarbon dating (1) and ancient genomics (2–4) have made significant contributions to our understanding of the disappearance of Neanderthals. However, Hominin remains are extremely rare and limit the impact of these methods.

There are currently very few known remains of late Neanderthals and early modern humans across Eurasia. A significant proportion of the faunal remains excavated at Paleolithic sites are not identifiable using traditional zooarchaeological methods (5) This is mostly due to the activity of carnivores (6), anthropogenic fragmentation (7), postdepositional movement and erosion, and taphonomic influences (8). At some sites, the proportion of unidentifiable bones is more than 95% (e.g., Denisova Cave), resulting in substantial loss of archaeological information. Collagen peptide mass fingerprinting, also known as Zooarchaeology by Mass Spectrometry (ZooMS), enables the identification of unidentifiable bone fragments to species/genus level with a high rate of throughput (9, 10). This method has allowed archaeologists to explore the full spectrum of faunal remains from archaeological contexts and in some instances to identify rare hominin bone fragments (11–14).

**Fig. 1.**
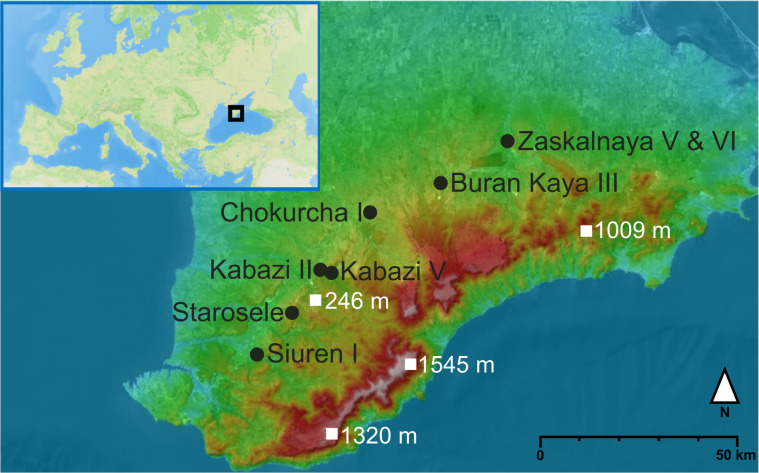
The location of the sites of Starosele, Siuren I, Kabazi II, Kabazi V, and Chokurcha in the Crimean Peninsula. The red to brown colors represent the first and highest mountain range, which reaches elevations over 1,000 m, the lighter and less frequent red to orange color indicates the second middle mountain range; due to low elevations, the lowermost third range in the North of the middle range is almost not visible in this map. Map made with Mapbox and Topographic map.

We used ZooMS to analyze Paleolithic bone assemblages from the Crimean site of Starosele (Fig. 1). The aims were threefold; first, to widen our understanding of the subsistence strategies of the human occupants, second, to screen bone fragments to find potential hominin bone remains which could be used for direct dating, genetic sequencing, and paleodietary analysis, and finally to determine the history of hominin occupation on the Crimean Peninsula.

## The Archaeological Site of Starosele.

The Crimean Peninsula contains numerous well-preserved, stratified Paleolithic sites, with many spanning within the transitional period in terms of bioculture and hominin occupations of approximately 47,000 to 42,000 cal BP (15). Based on previous radiocarbon dates, the Crimean Peninsula has been described as a refugium for late surviving Neanderthals just before their disappearance. However, new research, including radiocarbon dating using compound-specific techniques, has shown that this may need to be modified because some of the previous dates determined are, in fact, much younger in age in comparison to the compound-specific results (16).

Starosele is a rock-shelter positioned within a steep canyon (17, 18) and comprises four distinct cultural layers (19). The stratigraphy and site plan are shown in *SI Appendix*, section A. Level 2 and 3 are separated by a rockfall event, with evidence suggesting that Level 4 was briefly occupied (17). Previously, human remains within Level 1 were assumed to be Medieval Muslim burials after many decades of debate and uncertainty (20).

The Crimean Micoquian lasted from the Last Interglacial until approximately 40 kyr cal BP (21, 22). This technocomplex is characterized by soft hammer bifacial thinning and retouching, to produce bifacial tools (23). Micoquian lithic assemblages are exclusively linked with Neanderthals across Eurasia, as evidenced by the lithic assemblages co-occurring with Neanderthal remains at various sites, such as Kiik Koba (24), Zaskalnaya VI (25), Stajnia Cave (26), Mezmaiskaya (27) Chagyrskaya and Okladnikov (28, 29). In Starosele, Levels 1, 2, and 4 are assigned to the Crimean Micoquian, while Level 3 remains unassigned to a previously known lithic industry (Starosele-Level.3-Industry). Bifacial tools are present at all levels, except Level 3, which exhibits blank production methods (30). This may indicate that another group of Neanderthals was present at the site at this time. However, further analysis is needed to characterize the lithic assemblage fully.

Zooarchaeological analyses have previously been conducted at Starosele. A significant percentage of the faunal spectrum could not be identified to species through morphological inspection alone. Of the identified fauna, *Equus hydruntinus* dominates the assemblages. Level 1 comprises ~75% Equidae (19, 31). Cervus sp. (deer), various carnivores, and several bird species have also been identified in all levels (31, 32). Many of the faunal remains exhibit anthropogenic cut marks, indicating that butchering activities occurred at the site. Level 1 has a large assemblage of faunal material, with extensive evidence of animal butchery. However, there is also a high degree of fragmentation among the identified remains, which include *Sus* sp., *Vulpes* sp., *Ursus* sp., *Crocuta* sp., *Rupicapra rupicapra*, Cervid, Elephantidae, *Bos/Bison,* and *Saiga* sp. (17).

Here we investigate the potential of the fragmented faunal assemblage for hominin bones and apply paleoproteomic analyses to unidentified fauna from Starosele. Following this, we apply DNA and radiocarbon analyses to a small number of bones.

## Results

### Paleoproteomics.

On the basis of morphological and preservation characteristics, i.e., unburnt, sizeable bones over 2 cm long, we applied ZooMS to 150 bone fragments from all layers of Starosele. For these, we obtained 146 ZooMS identifications (97.3% success rate) (*SI Appendix*, section C and Dataset S1). The vast majority of these were identified as animal bones. Overall, the ZooMS results broadly confirm what was known from formal zooarchaeological analyses (17, 32) with six taxa forming the bulk of the identifications and *Equus* sp. dominating (93%). However, within Level 3, Square F21, a higher percentage of Bovidae or Cervidae/Bovidae is observed in comparison to the other stratigraphic contexts. Two faunal species were found only using ZooMS; Rhinocerotidae and *Canis lupus*. The taxonomic identifications are consistent with a classic MOIS3 paleoenvironment (33), characterized by fluctuating warmer and colder environments during stadial and interstadial episodes (34).

### Starosele Human Remains.

**Fig. 2.**
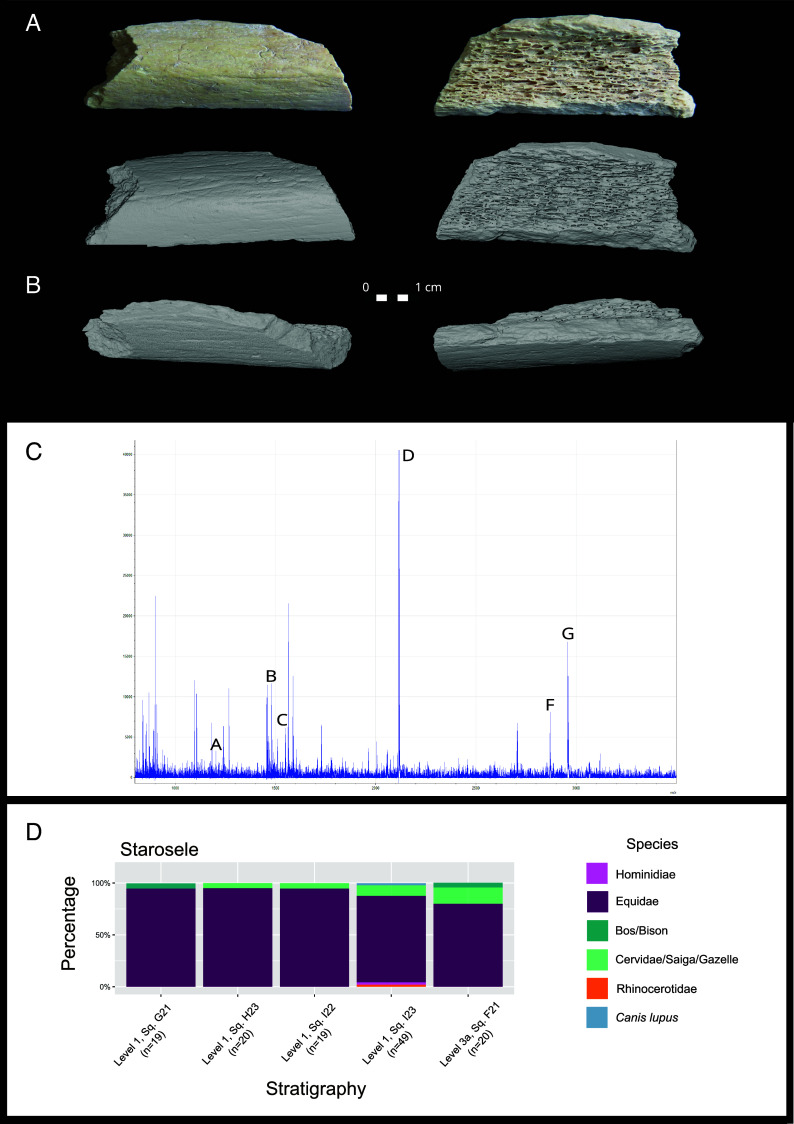
(*A*) Photograph of the hominin bone, Star 1, from Level 1, Square I23 (Z00113) shortly after it was identified using biomolecular methods. (*B*) µCT scans of the hominin bone. (*C*) MALDI mass spectrometry data from Z00113, Star 1. Previously published peptide markers are labeled A–D and F and G. Peptide marker E is missing. (*D*) Summary of the ZooMS results from Starosele plotted by the stratigraphic context and percentage. The exact percentage for each taxon can be found in *SI Appendix*, section C.

**Fig. 3.**
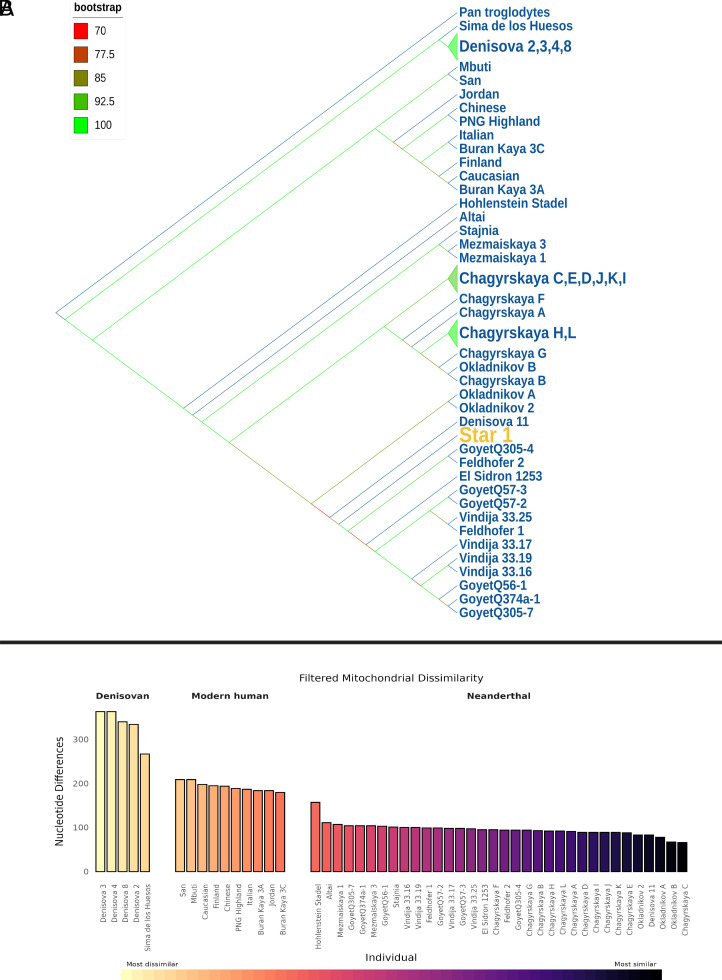
(*A*) Phylogenetic tree of the mitochondrial genome featuring Star 1, together with other ancient hominins and modern humans. The Starosele, Star 1 branch, is highlighted in orange, and the bootstrap values are marked in a color gradient from red (minimum) to green (maximum). For the actual bootstrap values, see *SI Appendix*, section D. (*B*) Number of pairwise differences between Star 1 and other ancient and modern humans, sorted from most to least dissimilar.

Among the ZooMS-analyzed bones, we identified a Hominidae bone fragment (Fig. 2 *A* and *B*), which originated from Level 1 (Micoquian), Square I23 (*SI Appendix*, section A), close to previously identified human burials (see below). We refer to this new specimen as Star 1. See *SI Appendix*, section B for stable isotope information. The ZooMS spectra for Star 1 (coded Z00113) shows the six peptide markers (A-D & F and G) associated with this Family, including 1,235.71, 1,478.74, 1,580.86, 2,115.19, 2,869.56, 2,957.68 (Fig. 3*C*) (35, 36). Peptide marker E is missing, which may be due to collagen degradation and its scarce presence in humans (12). The human bone has no clear recognizable morphological characteristics or human-induced modifications, which is why it was overlooked during excavation and zooarchaeological analysis (Fig. 2*A*).

The fragment was microcomputed tomography (µCT) scanned and virtually analyzed prior to any further invasive examinations. It measures 49.8 mm in maximum length and 18.8 mm in maximum width. Two relatively flat surfaces meet at an approximately orthogonal angle (Fig. 2 *A* and *B*). The fragment exhibits minimal signs of fossilization and is composed primarily of cortical bone, with residual trabecular bone lining the entire inner surface. Cross-sectional measurements of the cortical bone yielded a maximum thickness of 6.21 mm at the angled midsection. The thickness gradually decreases along both surfaces extending from the corner, with the thinnest part measuring 2.78 mm. According to Gosman et al. (2013) (37), the individual to whom this fragment belonged was likely not younger than an adolescent. The surface data can be found at the following link https://www.virtual-anthropology.com/3d-data/free-data/.

Based on the size, shape, and curvature of the fragment, as well as its ZooMS classification, we suggest it could only originate from a hominin femur or humerus. According to the comparative analysis we performed, the fragment most likely derives from the anterior distal shaft of a femur, since it closely matches our comparable specimens in cross sectional angle, continuity of the surfaces, and cortical thickness, while it does not align with humeri with respect to these characteristics. However, it should be noted that these results are based on a small comparative sample.

Radiocarbon dating was undertaken on the bone using the standard ultrafiltration approach at the University of Vienna’s Higham Lab. The result was 39,858 ± 736 BP (VIE-1203). The calibrated age ranges between 43,860 to 42,690 cal BP (at 68.3% prob,) and 44,390 to 42,450 cal BP (at 95.4% prob.), a result consistent with previous radiocarbon dates from Level 1 (*SI Appendix*, section A). We also treated the collagen from the bone sample following ultrafiltration with acid hydrolysis and purified the amino acids using XAD-2 resin chromatography. This resulted in a radiocarbon date of 43,212 ± 295 BP (VIE-1541). The calibrated age ranges between 45,910 to 45,340 cal BP (at 68.3% prob,) and 46,130 to 44,990 cal BP (at 95.4% prob.). We conclude that the XAD radiocarbon date is the most accurate of the two, as it has been shown in the past to remove environmental and museum-derived contaminants successfully and thereby yield more accurate results compared with bulk collagen determinations (38, 39).

The bone was processed for DNA extraction and sequencing and two libraries were prepared, using 40 mg (STS1.1) and 10 mg (STS1.2) of bone powder, respectively (40). One of the subsamples (STS1.2) was subjected to a bleach pretreatment (41). Two single-stranded DNA libraries were constructed using an optimal method for ancient DNA (42). Diagnostic positions for derived Neanderthal alleles compared to modern humans (43) indicated that the sample contains DNA matching the Neanderthal allele (diagnostic positions with Neanderthal reads: 10/22 for STS1.1 and 11/52 for STS1.2). Due to the extremely low endogenous DNA content in the constructed libraries (0.038% STS1.1; 0.043% STS1.2), we performed mitochondrial target enrichment, resulting in an average coverage of 2.28-fold for the mitochondrial genome.

This mitochondrial genome confirms that the bone is most consistent with belonging to the Neanderthal lineage, not modern human or Denisovan-like lineages (44–46). Although due to relatively low coverage the placement in a phylogenetic tree cannot be fully resolved, Star 1 appears basal to European Neanderthals (GoyetQ305-4/Feldhofer2), and derived to Siberian Neanderthals (Denisova 11, Okladnikov A, 2). When comparing the mtDNA sequence to complete Neanderthal mtDNA data (Fig. 3*B*), Star 1 appears most closely related to five hominins from three sites; Denisova 11, Chagyrskaya E, and Okladnikov 2, A and B (12, 47, 48). Notably, all three are in the Altai region of Russian Siberia despite being ~3,000 km distant.

## Discussion

The Neanderthal bone fragment identified in this work is a key finding in the light of previous research conducted at Starosele. The site was known for many decades for the discovery of the widely cited “Starosele child” in the 1950s, originally excavated by Formozov (32). The remains, estimated to be of an 18 to 19-mo-old child, have been controversial and strongly debated. They were initially identified as a “transitional Neanderthal” or *H. sapiens* individual by Formozov and others, who saw similarities with the remains found in the Levantine site of Skhul (32). Howell agreed, comparing the Starosele child to Skhul, Qafzeh, and modern humans in Russia (49–51). Later, in the 1990s, Soffer suggested that the child was an anatomically modern human (19). Confusion regarding biological identification was mirrored by uncertainty in the age of the remains and their context, owing to excavation methods that were not of a robust nature and a lack of photographs or detailed field notes that could confirm the exact positioning of the remains (19). New excavations by a joint Ukrainian/American team between 1993 to 1995, however, showed that it was most likely to be a much later burial, perhaps late Medieval (19). Marks and Chabai found two other burials at the site in 1993 to 1994: one infant and one adult, in squares H25 (adult) and I22 (infant). They suggested that these were probably 17/18th century Muslim burials, based on the stratigraphy of the burial pits, layout of the bodies and their direction and the typology of the ceramic pieces from the burial pits. Hence, it was inferred that the Starosele child burial was probably from a similar period (*SI Appendix*, section A) (19). No dating was performed on the new human remains, however, and while it would be interesting to demonstrate their late age, it has not been possible to do this in the current study. The most likely explanation is that the burials are late and were interred into Middle Paleolithic cultural horizons (Level 1), where the Star 1 human bone originates.

Level 1 has been dated using various methods and protocols (*SI Appendix*, section A). The calibrated ages of the latest three radiocarbon dates using the ultrafiltration protocol from Level 1 range between 47,710 to 42,490 cal BP (at 68.3% prob,) and 51,010 to 44,280 cal BP (at 95.4% prob.) (*SI Appendix*, section A). New compound specific protocols are currently being applied to date other bones.

Despite the extremely low endogenous DNA content and the lack of nuclear DNA, the 2.28-fold coverage after targeted capture shows that Star 1 did not belong to the divergent lineage of Hohlenstein Stadel (52). The sequence instead places it as being most similar to Denisova 11, Chagyrskaya E, and Okladnikov 2, A and B, which are all in the Altai region (Fig. 3). Because the genome data are incomplete, especially in parts of the D-loop and the hypervariable regions, we cannot confirm whether Star 1 can be assigned to this lineage of Siberian Neanderthals or represents a sister lineage. However, in the currently available record of Neanderthal diversity, the Siberian Neanderthals are the closest related individuals. Okladnikov and Chagyrskaya have been described as short-term, seasonal hunting camps, occupied by Neanderthals (47, 53). One suggestion, based on the close affinity of these Neanderthal mitochondrial genomes, is that they might represent an expansive movement of a Neanderthal group, most likely in an easterly direction, at some point during MIS4 ranging from ∼71 to 57 ka (28).

**Fig. 4.**
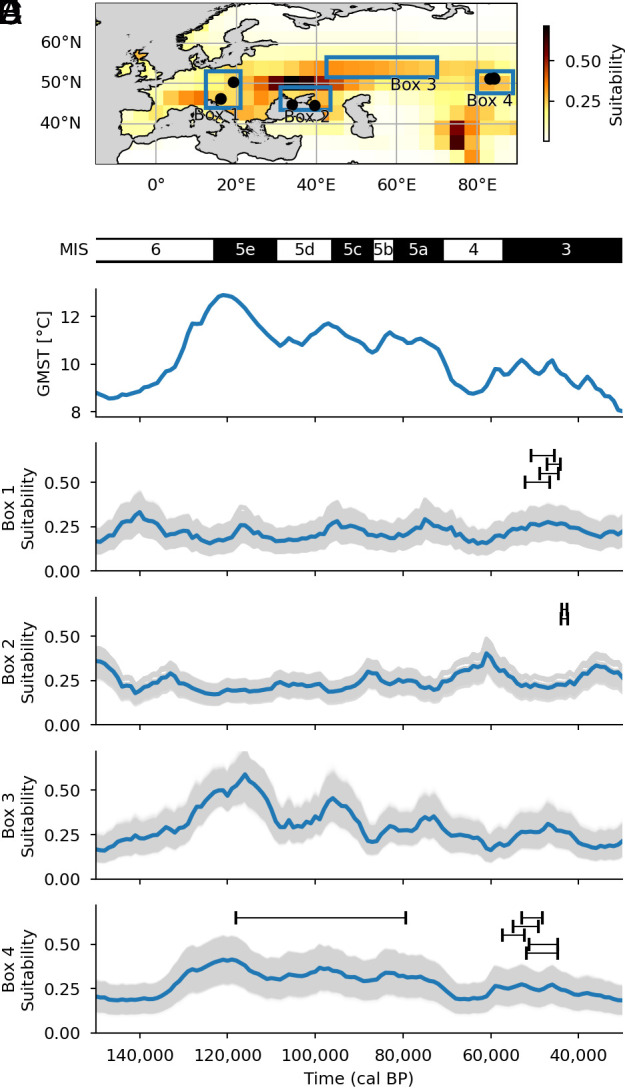
Average habitat suitability of Star 1 and genetically related hominins from Croatia, Poland, the Crimean Peninsula, and Altai region (*A*). With time series of global mean average temperature from the 3 My transient CESM1.2 simulation (61, 62) (*B*), and results of the habitat suitability model over the area in Box 1, 2, 3, and 4 (*C*–*F*) where the blue line is the average suitability and the gray lines are the results of the individual models of the Monte Carlo analysis (*Methods*). In box 1,2, and 4 each sample included in the primary habitat suitability model was added with the entire time uncertainty (black bars) and at the top of the timeline plots the marine isotope stages (MIS) are indicated in black and white, representing warmer and colder periods, respectively.

To demonstrate further potential dispersal corridors and identify paleoclimatic and paleoenvironmental preferences, we constructed two Mahalanobis distance habitat suitability models covering the window between MIS6-MIS3, ~150 to 30,000 BP (*Methods*). This method has previously been used to explore habitat overlap between Neanderthals and Denisovans (54). Our habitat suitability models suggest that climatic conditions linking central Eurasia (Altai) and eastern Europe were most favorable during interglacial periods, supporting MIS 5e as a potentially attractive time for dispersal along this route [Fig. 4 and *SI Appendix*, section F, (55)]. We identified the most likely route between the Crimean Peninsula and Altai region along longitude 55°N (Fig. 4 *A* and *E*). This dating estimate appears to fit the evidence in the Altai for the presence of Neanderthals at Denisova Cave, where genetic and chronometric evidence places them between ~130,000 to 100,000 y ago. There are hints that Neanderthals might have been present prior to this, based on mtDNA in ~170,000 y ago sediment at Denisova Cave, but no human remains of this antiquity have been found yet (56). Later, Neanderthals appear at sites such as Okladnikov Cave. The initial radiocarbon dating of Neanderthal remains there disclosed a wide range of results, including ages younger than 40,000 cal BP. Okladnikov 2, 11, and 15 have been radiocarbon dated using robust single compound hydroxyproline dating to older than 42,000 cal BP (57, 58). At other Altai sites, such as Chagyrskaya, the most likely age of the Neanderthals is more precise and dates to between 59 and 51,000 cal BP (47). This suggests two, perhaps three, dispersals of Neanderthals from the west to the Altai at 170, 120-100, and ~60,000 cal BP. This hypothesis is supported by genetic data which suggest that the Chagyrskaya and Okladnikov Neanderthals were more closely related to the Vindija Cave 33.19 individual (59), than to the earlier Altai Neanderthal. Similarly, Denisova 11’s Neanderthal mother is also genetically more closely related to European populations of Neanderthals, represented by Vindija 33.19, having 19.6% alleles present compared with 12.4% in Altai Neanderthals (60). It seems most parsimonious to conclude, therefore, that movement occurred from west to east and during times of favorable climate, particularly during MIS5e. Potential later movement, however, accounting for the Chagyrskaya and Okladnikov Neanderthals, seems more likely to have occurred during less benign climate phases, perhaps during MIS4.

In addition to the genetic links between Starosele, Chagyrskaya, and Okladnikov, these Neanderthal groups appear to have had similar subsistence strategies, focused upon exploiting mainly horses, as well as bison and small ungulates, characteristic of steppe and piedmont environments. Sites dating to these periods have been interpreted as hunting and butchering sites focusing on large herbivores and ungulates within steppe environments (23, 63). Lithic assemblages excavated from Okladnikov, Chagyrskaya, and Starosele are also very similar, and characterized by artifacts of the Micoquian technocomplex (22, 28, 64, 65). A statistical study examining the lithic components of Micoquian assemblages from Chagyrskaya and comparing them to other Micoquian assemblages, showed that the Crimean Micoquian was significantly closer to Chagyrskaya than Denisova (64). The lithic assemblages from Chagyrskaya, in turn, compare closely to Okladnikov assemblages, being a unique variant in the Altai region, possibly representing small Neanderthal groups (63). The Neanderthals at Denisova Cave had a completely different lithic assemblage, indicating a different population of Neanderthals were present, and at a much earlier time (29, 66).

We may conclude, from the archaeological and genetic evidence, therefore, that it is likely that Neanderthals were in the Altai at two, perhaps even three, different periods. Our results show that Starosele was part of a wide-ranging dispersal network of Neanderthals, linked by genes, lithics and behavior.

## Methods

### Sample Selection.

All faunal remains were excavated between 1993 to 1994 and sampled by EP at the University of Erlangen–Nuremberg, Germany. The samples were collected for ZooMS analysis depending on the availability and layers present at the institution. Approximately 20 to 25 bones were selected from each layer/level within different squares, when possible. Some bags containing the faunal remains had a large number of bones (50 to 100), while others contained approximately 20. A further 50 bones were sampled from Level 1, square I23, after the hominin bone was found (Table 1). Star 1 originally weighed 6.66 g, measuring a maximum 52.3 mm in length and 16.5 mm in width. In total, 26 mg was taken for ZooMS analysis, with ~900 mg taken for radiocarbon dating. Stable isotope analysis was undertaken on collagen from the radiocarbon dating preparation.

**Table 1.** Number of bones analyzed for ZooMS analysis from five different square numbers from Levels 1 and 3

Site	Typology	Level	Square	No. of bones
Starosele	Starosele-Level-3-Industry	3	F21	20
Starosele	Micoquian	1	G21	20
Starosele	Micoquian	1	H23	20
Starosele	Micoquian	1	I23	70
Starosele	Micoquian	1	I22	20

### Sampling.

The bone samples were manually cleaned with air abrasion mixed with aluminum oxide powder at the Douka/Higham labs, Department of Evolutionary Anthropology at the University of Vienna, Austria. This enabled the removal of superficial contaminants before sampling, with around 30 mg of bone being clipped off with clean dental pliers for ZooMS analysis. For the human bone, 26 mg was clipped for ZooMS and ~900 mg sampled for radiocarbon dating.

### CT Scanning.

The Star 1 hominin bone was µCT scanned at the Vienna Micro-CT Lab with a VISCOM X8060 Scanner (39 μm, 110 kV, 400 µA, 1,400 ms, 0.75 mm copper filter), before further invasive sampling for dating and paleoproteomics were undertaken. For further comparative analysis a femur and humerus of one Sub-Saharan African and one European individual respectively were scanned at the same facility (63 µm, 130 kV, 320 µA, 1,400 ms, 0.75 mm copper filter). Additionally, scans of the Neanderthal 1 specimen from Feldhofer Grotto were provided from the Max Planck Institute for Evolutionary Anthropology Leipzig with a resolution of 91 µm. All data were virtually analyzed and measured in the Amira 3D 2024.1 software from Thermo Fisher.

### ZooMS.

The ZooMS analysis was conducted at the Douka Lab, Department of Evolutionary Anthropology, University of Vienna, Austria. Bone samples were analyzed using an acid insoluble protocol (9, 67). Following wet chemistry, the spectra were analyzed using flexAnalysis 3.4 (Brucker Daltonics) and mMass software (68). Subsequently, the spectra were compared against known peptide markers in a reference library (9). Some species/family faunal remains had already been identified from previous zoological analyses.

ZooMS analysis was undertaken using the acid insoluble collagen protocol (9). The bone specimens were first sampled using pliers, chipping off 15 to 25 mg of bone. The samples were demineralized up to 48 h with 500 μL of cold (4 °C) 0.6 M HCl (covering the sample), until somewhat spongy. After demineralization, samples were vortexed for approximately 10 s and centrifuged for 1 min at 12 RPM. The samples then went through 2 to 3 washes with 200 μL of 50 mM AmBic, followed by centrifuging and discarding the AmBic solution. Following this, 100 μL of 50 mM AmBic (ammonium bicarbonate buffer, pH 8.0) was inserted into each sample and then incubated for 1 h at 65 °C for gelatinization. Half (50 μL) of the sample was removed in case a backup was needed in the future. Trypsin digestion was undertaken by inserting 1 μL of trypsin solution 0.4 μg (Thermo Scientific Pierce™ Trypsin Protease), which was added to each supernatant and digested for 18 h at 37 °C. The digested samples were treated with 1 μL of 5% Trifluoroacetic Acid (TFA) to stop further enzymatic digestion. ZipTipping was performed for purification and peptide extraction, using C18 ZipTips (Thermo Scientific Pierce™ C18 Tips), using 50 mL of 50% ACN in 0.5% TFA. A calibrant at 1:2 ratio of 0.5 μL was added to the plate, with 0.5 μL matrix solution. The samples (0.5 μL) were spotted with 0.5 μL of matrix solution. Thereafter, the processed samples were analyzed using a Bruker Autoflex Speed Matrix-Assisted Laser Desorption/Ionization Time-of-Flight (LRF MALDI ToF/ToF) mass spectrometer (69). The peptide marker (A-G) was manually inspected in mMass 5.5.0 (70).

After manual taxon identification, SpecieScan software was used to confirm taxon identifications (71). SpecieScan is a semiautomated process using R and Python to output correlated identifications from reference databases.

### Radiocarbon Dating.

Collagen extraction and pretreatment of the bone was conducted at the Higham Lab, Department of Evolutionary Anthropology, University of Vienna, Austria. The human bone underwent ABA (Acid–Base–Acid) and ultrafiltration protocols (72, 73). The bone was first demineralized with 0.5 M HCl twice for 2 h and then for ~24 h. Then the sample was washed three times with ultrapure Milli-Q water. Following this, a base wash was performed with 0.1 M NaOH for 30 min. Three more washes with Milli-Q water were then undertaken, with a 15-min treatment of 0.5 M HCl, followed by three Milli-Q water washes. The collagen was then gelatinized in pH3 water for 20 h at 75 °C. The gelatin was then filtered with a precleaned Ezee-filter, before being ultrafiltered using a precleaned Vivaspin^®^ Turbo 15 30 kD MWCO and centrifuged until 1.0 mL of the >30 kD gelatin fraction remained (~20 min at 2,700 RPM). The gelatin was then placed into a clean glass tube with Milli-Q water and frozen before being freeze-dried for 48 h.

After freeze-drying the collagen yielded 24 mg of collagen. A subsample of the collagen was weighed into precleaned tin capsules then combusted and graphitized using an AGE3 system, before being accelerator mass spectrometry (AMS) dated at VERA, University of Vienna.

XAD-2 resin treatment was also undertaken on a subsample of the leftover collagen (8.33 mg) after freeze-drying. This method involves hydrolysis of the collagen into amino acids and their recovery using ion-exchange columns (74). The sample was hydrolyzed for 24 h at 110 °C, in 6 M HCl. A precleaned and preconditioned XAD column was used to allow the passage of the hydrolyzed amino acids. The column had a pre-fitted filter frit at the bottom, and a filter frit was placed above ~100 µL of XAD-2 resin. To clean the resin, 20 washes of 1 M HCl and 10 washes of 6 M HCl were passed through the column. Then the sample passed through the column, with one more wash of 1 mL of 6 M HCl, to collect any leftover amino acids. The sample was then placed into a Genevac system for 12 h, to remove the HCl acid through evaporation. ~100 µL of Milli-Q water was placed into the tube and then placed back in the Genevac for 1.5 h until full evaporation to remove trace remnants of HCl that might otherwise interfere with the graphitization process later. Following this, ~100 µL of Milli-Q was placed again into the sample tube to collect the sample, before transferal of the sample into a precleaned tin boat together with precleaned chromosorb for combustion, graphitization, and AMS dating.

OxCal v. 4.4.3 software (75) and the INTCAL20 calibration curve (76) was used to calibrate the result.

### Stable Isotopes.

After the collagen was weighed out for radiocarbon dating, 300 μg was weighed into a tin capsule for isotopic analysis of carbon and nitrogen by elemental analyzer-isotope ratio mass spectrometry (Thermo Scientific EA-Isolink with a Flash 2000 coupled to a Delta V Advantage isotope ratio mass spectrometer) in the Silver Laboratory (Large-Instrument Facility for Advanced Isotope Research) at the Center of Microbiology and Environmental Systems Science of the University of Vienna. Stable isotope values of all samples are measured relative to the laboratory standard alanine, which has a reproducibility of 0.07 wt% for N and 0.1 wt% for δ^13^C and δ^15^N values are reported relative to the Vienna Pee Dee belemnite and Atmospheric air standard, respectively, and are measured with an analytical precision of ± 0.1‰ for both δ^13^C and δ^15^N values.

### DNA Extraction, Library Construction, and Target Enrichment.

Ancient DNA extraction was carried out in the Pinhasi Lab, with dedicated clean-room facilities at the University of Vienna as per the protocol first described by Dabney et al. (2013) (40). Two bone powder samples were obtained using a dental drill and incubated overnight (18 h) at 37 °C on a rotation plate (800 rpm) in 1 mL of extraction buffer (final concentrations: 0.45 M Ethylenediaminetetraacetic acid, 0.25 mg/mL proteinase K). The supernatant was mixed with 13 mL of binding buffer (final concentrations: 5 M guanidine hydrochloride, 40% [v/v] isopropanol, 0.05% Tween-20, and 90 mM sodium acetate) in preassembled silica spin columns (Roche) following Korlević et al. (2015), (41). Following a 4-min centrifugation at 1,500 rpm, the columns were disassembled. The remaining spin columns were washed twice with 650 μL of PE purification wash buffer (Qiagen; in-house) with an intermediate centrifugation step at 6,000 rpm, followed by a dry spin. DNA was eluted in 50 μL EBT (Qiagen with 0.05% Tween-20 added) following a 5-min incubation at 37 °C and centrifugation at maximum speed for 1 min. The DNA concentrations were measured via the QuBit dsDNA DNA High Sensitivity kit. Blank samples at the beginning and the end of the batches were included to control potential cross-contamination.

Single-stranded libraries were built as per Kapp et al. (2021) (42). Based on initial DNA concentration, appropriate dilutions of single-stranded DNA binding protein (SSB), P5 and P7 adapter dilutions were prepared. 20 μL of DNA extract were mixed with 2 μL of the appropriate SSB dilution. The mix was incubated at 95 °C for 3 min and transferred onto an ice bath. While on ice, ×1 μL of the appropriate dilutions of P5 and P7 adapter dilutions were added together with 26 μL of the Santa Cruz Reaction mix. An incubation at 37 °C for 45 min followed. MinElute columns and buffers from the Qiagen MinElute PCR Purification kits were used for intermediate clean-up steps. qPCR was performed on 1:40 dilution of the resulting libraries in order to estimate the number of amplification cycles to take place post unique molecular indexing (UMI). UMIs from the Ancient Meyer collection were used to double-index the samples, following an amplification with the NebNext Q5U Master Mix DNA Polymerase (NEB). Indexed libraries were pooled with other samples and submitted to the Vienna Biocenter Core Facility for Next Generation Sequencing (VBCF NGS) where the samples were sequenced on a single lane of an Illumina NovaSeq X Plus single-read mode 100 bp. For mitochondrial enrichment we used the myBaits Expert Mito kit (Arbor Biosciences), following the protocols recommended by the provider.

### Bioinformatic Analysis.

Standard Illumina adapters were cut with leeHom v.1.2.18 in –ancient-dna mode and filtering for sequences longer than 30 bp was performed with seqtk (77). Mapping to hg19 and Revised Cambridge reference sequences was performed with bwa aln v. 0.7.18 with ancient DNA settings (*SI Appendix*, section E). Unmapped reads were removed with samtools v.1.20 and PCR duplicates were filtered out with GATK MarkDuplicates v.3.1.1. Typical Ancient DNA deamination patterns were confirmed with MapDamage v.2.2.2, contamination was assessed with schmutzi v.1.5.7 and filtering out of nondeaminated reads was performed with PMDtools python3 updated version (78–80). Mitochondrial genome sequence and quality was confirmed with samtools, mosdepth, and qualimap, and visualized with BamPlotter (81–83). A consensus sequence was called with ANGSD –doFasta (*SI Appendix*, section E) and multiple sequence alignment including a collection of present-day humans, Neanderthals, and *Pan troglodytes* was performed via the MEGA11 in-built MUSCLE algorithm (84, 85). The phylogenetic tree was constructed with IQ-tree (*SI Appendix*, section E) and visualized with Interactive Tree of Life (86, 87). Pairwise nucleotide differences between Star 1 and other genomes in the alignment were calculated using a custom Python script available on the GitHub page of the project. All code used is available at https://github.com/KonstantinaChe/Star1_Neanderthal.

Raw untrimmed sequencing data are available at the European Nucleotide Archive (ENA) under the accession number: PRJEB93921. All code used for data processing is available at https://github.com/KonstantinaChe/Star1_Neanderthal.

### Mahalanobis Distance Habitat Suitability Modeling.

Two Mahalanobis distance habitat suitability models were constructed to assess the ecological connections between three geographic regions: Croatia/Poland, the Crimean Peninsula, and the Altai region.

The primary model utilized only dated fossil data, comprising a limited dataset of 13 samples (Table 2). To assess the validity of the primary model, a second, more comprehensive model was developed. This validation model incorporated data from an extensive fossil and archaeological database (47, 48, 57, 66, 88) focused on the regions surrounding the sample locations of the primary model. The following coordinate boundaries were used for the geographical extent of these regions:

**Table 2.** Specimens and dated materials including their time uncertainty used for constructing the primary habitat suitability model

Specimen/dated material	latitude	longitude	min_age	max_age	Location	Age estimate method
Vi-33-19	46.301562	16.079687	50,901	45,561	Box 1	Age at 68.3% probability
Vi-208	46.301562	16.079687	47,256	44,126	Box 1	Age at 68.3% probability
Vi-207	46.301562	16.079687	48,904	44,579	Box 1	Age at 68.3% probability
Mammoth from layer D - same layer as the discovered Neanderthals OxA-24944	50.366	19.294	52,257	46,646	Box 1	Age at 68.3% probability
Star 1	44.75	33.92	43,858	42,695	Box 2	Age at 68.3% probability
Mezmaiskaya 2	44.5540	39.5534	43,986	42,526	Box 2	Age at 68.3% probability
Chagyrskaya Level 6a where Neanderthal remains were found	51.263299	83.091628	53,000	48,200	Box 4	Weighted arithmetic mean age from four samples
Chagyrskaya Level 6b where Neanderthal remains were found	51.263299	83.091628	55,000	49,200	Box 4	Weighted arithmetic mean age from two samples
Chagyrskaya Level 6C where Neanderthal remains were found	51.263299	83.091628	57,400	52,400	Box 4	Weighted arithmetic mean age from nine samples
Layer 7 sediment where a Neanderthal was found	51.40	84.20	51,338	44,716	Box 4	Age at 68.3% probability
Layer 7 sediment where a Neanderthal was found	51.40	84.20	51,903	44,801	Box 4	Age at 68.3% probability
Denisova 11 age estimates	51.235	84.403	118,100	79,300	Box 4	

Croatia/Poland: longitude: 12.5°E-21°E, latitude: 44°N-53°N

Crimean Peninsula: longitude: 31°E-43.5°E, latitude: 43.5°N-49°N

Altai region: longitude: 80°E-89°E, latitude: 47.5°N-53°N

For both models, we employed a Monte Carlo sampling approach to address two key challenges: 1) the uneven distribution of data points at the three locations and 2) the variable temporal uncertainties associated with the data.

For each ensemble member, we randomly sampled an equal number of samples from each location to ensure balanced spatial representation. The primary model utilized two samples per location (totaling six samples per ensemble member), while the validation model used 10 samples per location (totaling 30 samples per ensemble member). Additionally, for each sample, a random age was selected from within the age uncertainty.

Sampling the data using this approach ensured that we account for the uneven sample size and varying length in age uncertainty without weighting specific locations or specimens more heavily. This sampling process was repeated 5,000 times.

For each ensemble member, we constructed habitat suitability models by extracting 1,000-y averages of annual mean temperature (*T*) and precipitation (*P*) from a 3 My quasi-transient climate simulation at a 5.75 × 3.75 resolution (61, 62). The extracted temperature and precipitation correspond to the nearest spatiotemporal point for each sample in the ensemble member constructed using the Monte Carlo sampling approach. Environmental conditions across the selected points were concatenated into a single matrix $C^{i}=\left[T\left(Z^{i}\right),P\left(Z^{i}\right)\right]$, and used to compute the mean vector $\overset{-}{C^{i}}$ and inverse covariance matrix $\Sigma^{-1}$ of the sampled conditions. Mahalanobis distance was then calculated across the entire climate grid using these statistics $d_{M}=\sqrt{{\left(C^{i}-{C^{i}}^{\prime }\right)}^{T}\sum^{-1}\left(C^{i}-{C^{i}}^{\prime }\right)}$. Suitability was derived as the exponential decay of Mahalanobis distance, i.e., $\exp(-d_{M})$, where lower distances result in higher suitability.

## Supplementary Material

Appendix 01 (PDF)

Dataset S01 (CSV)

## Data Availability

Raw untrimmed sequencing data have been deposited in Star_1 Neanderthal (PRJEB93921–https://github.com/KonstantinaChe/Star1_Neanderthal) (89). Anonymized surface scan data have been deposited in Star 1 (https://www.virtual-anthropology.com/3d-data/free-data/) (90).
