# Supplementary material for: A new late Neanderthal from Crimea reveals long-distance connections across Eurasia
Source: Proc Natl Acad Sci U S A. 2025 Oct 27;122(45):e2518974122. doi: 10.1073/pnas.2518974122 (PMC12625898; doi:10.1073/pnas.2518974122)
Supplement: Supplementary file 1 — Appendix 01 (PDF) [file pnas.2518974122.sapp.pdf]

PNAS

Supporting Information for
A new late Neanderthal from Crimea reveals long distance connections across Eurasia

Emily M. Pigott[1,2*], Konstantina Cheshmedzhieva[1,2*], Elke Zeller[3] ,Laura van der Sluis[1,2], Manasij Pal Chowdhury[1,2], Maddalena Gianni[1,2], Emese Végh[1,2], Thorsten Uthmeier[3], Victor Chabai[4], Marylène Patou-Mathis[5], 'Petra G. Šimková [1,2], Jana N. Voglmayr [1,2], Gerhard W. Weber[1,2], Ron Pinhasi[1,2], Axel Timmermann [7,8], Martin Kuhlwilm[1,2], Katerina Douka[1,2] and Thomas Higham[1,2]

1. Department of Evolutionary Anthropology, University of Vienna, University Biology Building, Djerassiplatz 1, A-1030 Vienna, Austria.
2. Human Evolution and Archaeological Science (HEAS) Network, Vienna, Austria.
3. Department of Geosciences, University of Arizona, 1040, Tuscon, Arizona, United States of America.
4. Institut für Ur- und Frühgeschichte, FAU Erlangen-Nürnberg, Kochstr. 4/18, D-91054 Erlangen, Germany.
5. Institute of Archaeology, National Ukrainian Academy of Science, Volodymyr Ivasiuk av. 12, 04210 Kyiv, Ukraine.
6. Muséum National d'Histoire Naturelle, Institut de Paléontologie Humaine,1 rue René Panhard, 75013 Paris, France.
7. IBS Center for Climate Physics, 46241, Busan, Republic of Korea.
8. Department of Climate System, Pusan National University, Busan, South Korea

*Joint first author

Corresponding Author: Emily Pigott and Tom Higham **Email:** emily.pigott@univie.ac.at and Tom Higham Email: thomas.higham@univie.ac.at

This PDF file includes:

Supporting text
Supplementary information sections A-F
Supporting figures S1-S6
Supporting tables S1-S4
Legends for Datasets S1
SI References

Other supporting materials for this manuscript include the following:

Dataset S1 – Excel file

Supplementary information A: Site background of Starosele

Chronology of the site

The first dates published for Starosele originate from the 1956 Formozov's excavations, from Levels 1 and 2. Level 1 was found above the slabs of exfoliated limestone. Level 2 was found above and between the slabs of the exfoliated limestone (1). The site plan with the areas from different years of excavations and human remains found can be seen in Fig. S1. Level 2 has been previously dated to 36,160 ± 1250 BP (OxA-4133), for the deposits above the rock fall (2). Later radiocarbon dates were undertaken from the 1993 excavations from level 1 (Fig. S2), dating to 41,200 ± 1800 (OxA-4775) and 42,500 ± 3600 (OxA-4887) with ion exchange protocol (2). Further dating was undertaken by Oxford University, with a radiocarbon date of 43,000 ± 1700 BP (OxA-37084) and 41,300 ± 1300 BP (OxA-37083) (3). The overall radiocarbon dates can be seen in Table S1. These dates were undertaken with the pre-treatment method of ultrafiltration, which separates low molecular weight (MW) components from the high MW fraction (>30 kD) This has been shown to improve the collagen quality as well as remove much of the contaminating substances (4), demonstrating why there may be a difference in radiocarbon dates with different protocols. However, radiocarbon dates will be undertaken with XAD protocol, to remove any possible contamination in the bone samples. Presently, the date of the new Starosele human remains fits well with the previous ultrafiltered radiocarbon dates. The lithic industry within Level 1 has been identified as Crimean Micoquian (CM), which fits well with the human remain being categorised as derived Neanderthal within the broader context of archaeological and lithic record (5, 6).

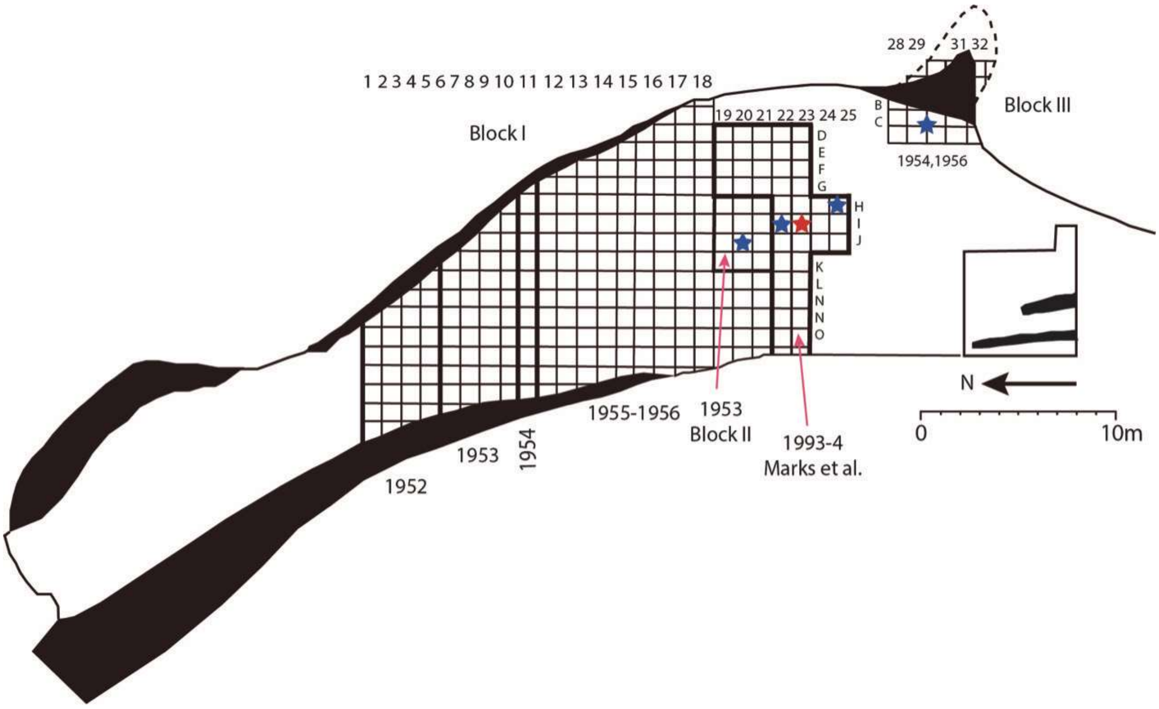

Figure S1. Excavation plan of the site of Starosele (after Marks et al., 1997, (7)). The black sections are solid rock mapped by Formozov. The thick lines denote the different excavation years undertaken by Formozov and the Joint Ukrainian/American Project (7). The blue stars indicate the position of the three previously identified burials at the site (the burial in J20 was the original child's burial), and the partial skeleton in Block III is also shown. Our new hominin bone is shown in red in square I23.

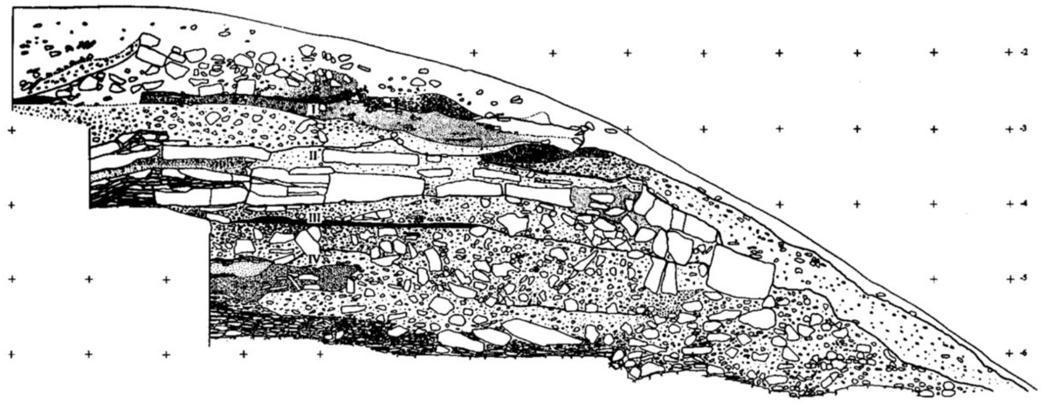

Figure S2. East/West profile at Starosele. From Marks et al. (1998), (8).

Table S1. Radiometric dates from Starosele. The dates used in the Bayesian model are highlighted in grey.

Lab No.	Material	Level	Pre-treatment method	Radiocarbon Age BP	Reference
U-series	Tooth	4	U-series	104,000 ± 8500 BP	**7**. Marks et al., (1997)
ESR LU	Tooth	3	ESR	42,000 ± 4700 BP	**7.** Marks et al., (1997)
U-series	Tooth	3	U-series	46,000 ± 2500 BP	**7**. Marks et al., (1997)
OxA-4133	Bone	Horizon 2, most likely Level 2	Unkown	36,160 ± 1250 BP	**2**. Hedges et al., (1996)
OxA-4775	Bone	1	Ion exchange	41,200 ± 1800 BP	**2.** Hedges et al., (1996)
OxA-4887	Bone	1	Ion exchange	42,500 ± 3600 BP	**2.** Hedges et al., (1996)
OxA-37084	Bone	1	Ultrafiltration	43,000 ± 1700 BP	**3.** Pigott et al., (2024)
OxA-37083	Bone	1	Ultrafiltration	41,300 ± 1300 BP	**3.** Pigott et al., (2024
VIE-1203	Human bone, Star 1	1	Ultrafiltration	39, 858 ± 736 BP	This paper
VIE-1541	Human bone, Star 1	1	XAD	43, 212 ± 295 BP	This paper

Supplementary information B: Stable isotopes

The stable isotope measurements of Z00113/VIE 1203 yielded a δ13C value of –19.3‰ and a δ15N value of 13.5‰. The carbon and nitrogen stable isotope values from this region for

humans can be compared to Buran Kaya III. The δ13C ratios range from –19.4‰ to –18.8‰ and the δ15N values range from 15.4‰ to 16.8‰ (9). When comparing the two sites, the stable isotope results suggest the hominin individual had a prominently carnivore diet. The δ13C value indicates the individual's protein source was from a terrestrial high trophic setting. In comparison, if the food source came from a marine environment, a δ13C value of around -12‰ would be expected (10). The nitrogen stable isotope value is somewhat high, which was excepted when looking at Neanderthals previously found in Europe. They tend to be top-level carnivores, including a range of large herbivores in their diet (9, 11). This can also be contrasted by the ZooMS results and previous zooarchaeological findings (12, 13). Maggots may have also been a source of nutrition, resulting in a possible higher nitrogen content (14). This may have allowed for a quick source of nutrition with a lower energy cost.

Supplementary information C: ZooMS

Since 2009, ZooMS has been used on materials such as bone, antler, ivory, leather, parchment and shells, which included studies from the Palaeolithic to the Medieval period (15, 16, 17, 18, 19, 20). The abilities of ZooMS are extraordinary, allowing thousands of fragmented bones to be analysed, which have never been assigned to a species/genus previously. One phenomenal example, which has proved to be pivotal, is the discovery of the Denisovans F1 hybrid "Denny" at Denisova Cave, Russia (21). This included nearly 10,000 bones being analysed (15). Most importantly, the method only requires a small amount of collagen for the method to be successful, allowing for low levels of destruction. There are also non-destructive protocols for more delicate or highly important artefacts such as ivory (22), bone (23) and parchment (19). However, as with all methods, there are some limitations. There is a possibility there will be a lack of collagen in the sample, or gaps in the reference database (24). Also, as expected, it is not possible to analyse cremated remains that were exposed to temperatures over 155°C with the current protocols (25). Furthermore, there are new methods appearing for spectra analysis to speed up the identification process. SpeciesScan is a semi-automated process which allows for species identification (26).

Overall, ZooMS is a powerful tool, which is rapidly expanding in protocols and types of material, in which the method can be successfully harnessed.

Starosele exhibits Equidae as the most prominent identified taxon (Table. S3). The prevalence of Equidae suggests an environment characterised by possible meadow-steppe with grassland conditions, which are generally the animal's preference, during the interstadials and stadial conditions (27). Additionally, the second most prominent taxon is Cervidae/Saiga/Gazelle and Bos/Bison. It is important to note that the differentiation between Cervidae/Saiga and Gazelle was not possible for all the samples. This was due to insufficient peptide preservation during spectra analysis. The co-existence of Bovidae or Cervidae suggests the presence of slightly open-steppe and possibly forest steppe environments (28, 29). The abundance of Equidae, Cervidae, and Bovidae, suggests significant hunting activity (13). The presence of Canis lupus may indicate the rock shelter was used for a den, which may have been likely for various carnivorous animals, to bring their prey to a place of safety. One fragment of Rhinocertidae was found within this study, which may indicate it was the prey of the humans occupying the rock shelter, or more likely of a wolf or hyena. We expect that if further ZooMS analysis was undertaken on a larger data set, more Rhinocerotidae faunal remains would be identified.

Table S2. The number of bone samples and their percentage within each square in Level 1 and Level 3a analysed.

**Species**	**Level and Sqaure number**				
	L.1, Sq. G21	L.1, Sq. H23	L.1, Sq. I22	L.1, Sq. I23	L.3a, Sq. F21

Hominidae	-	-	-	2% (n=1)	-
Equidae	94.7% (n=18)	95% (n=19)	94.7% (n=18)	85.5% (n=59)	80% (n=32)
Bos/Bison	5.3% (n=1)	-	-	-	5% (n=2)
Cervidae/Saiga/Gazelle	-	5% (n=1)	5.3% (n=1)	9.1% (n=6)	15% (n=6)
Rhinocerotidae	-	-	-	1.8% (n=1)	-
Canis lupus	-	-	-	1.8% (n=1)	-
Number of failed samples	1	-	1	2	-
Total in number	19	20	19	69	20

ZooMS has proven to be a very powerful tool in uncovering the subsistence strategies of Hominins in the Crimean Peninsula, particularly through the identification of the Neanderthal, Star 1. The discovery of human remains in Palaeolithic archaeological assemblages is exceptionally rare, highlighting the significant impact of ZooMS as an archaeological scientific method, especially when combined with radiocarbon dating and DNA analysis.

Dataset S1 (separate file): Spectra peak markers for all faunal and hominin samples in an excel file

Supplementary information D: DNA extraction, library preparation and sequencing

Ancient DNA extraction was carried out in the dedicated clean rooms at the University of Vienna. Two samples were obtained from the ZooMS-identified bone fragment and processed following the extraction protocol described by Dabney et al. (2013) (30), with modifications from Korlević et al. (2015), (31), (full description available at the Methods section). One of the samples also underwent a bleaching pre-treatment step, as outlined in Korlević et al. (2015) (31), (see Table. S3 for details).

Two single-stranded DNA libraries were constructed following the protocol of Kapp et al. (2021) (32). MinElute columns and buffers from the Qiagen MinElute PCR Purification Kit were used for intermediate clean-up steps. Quantitative PCR (qPCR) was performed using 1 µL of the original library to estimate the number of amplification cycles required post-indexing. The libraries were then double-indexed with unique molecular identifiers and amplified using the NEBNext Q5U Master Mix DNA Polymerase (NEB). Indexed libraries were pooled with other samples and sequenced on a single lane of an Illumina NovaSeq X Plus (read mode: (single read) SR100) at the Vienna Biocentre NGS core facility.

Table S3. Screening and post-capture information about the two Star 1 libraries we use in the study.

**Sample**	**STS1.1**	**STS1.2**
**Library Type**	single-stranded	single-stranded
**Bone powder input (mg)**	40	10
**Treatment factor**	bleach	-

**Endogenous DNA (%) post-screening**	0.03827	0.04304
**Endogenous DNA (%) post-capture**	0.140	0.070
**Contamination pre-PMD (%)**	49	80
**Contamination post-PMD (%)**	0-0.5	0-1.5

Screening data revealed extremely low endogenous DNA content, hence we performed mitochondrial enrichment using the myBaits Expert Mito kit (Arbor Biosciences), following the protocols recommended by the provider.

Supplementary information E: Bioinformatic Analysis

We analyzed sequencing data from two libraries: STS1.1 (5,405,843 reads) and STS1.2 (7,655,954 reads). We used leeHom (version 1.2.18) to remove adapter sequences, and performed mapping to the human genome build hg19 using bwa aln (version 0.7.18), with ancient DNA settings. Finally, unmappend reads were removed using samtools (version 1.20), and uniquely mapped reads were retained using GATK MarkDuplicates (version 3.1.1). In both libraries, endogenous content (as uniquely mapped reads divided by all produced reads) was low: 0.038% (STS1.1) and 0.043% (STS1.2), while duplication rates were high (75.6% for STS1.1 and 31% for STS1.2). These results suggested that further sequencing would not yield sufficient genome-wide coverage for population genetic analyses. However, this initial screening data was investigated for positions where almost all modern humans carry a derived allele (>99% frequency) and the high coverage Neanderthals carry the ancestral allele (33). Reads overlapping with these positions were queried using bcftools mpileup (34), analogous to a procedure applied to identify modern human contamination in non-human primates (35).

Using these diagnostic sites, we identify 22 (STS1.1) and 55 (STS1.2) reads overlapping such positions, among which 45% (STS1.1) and 23% (STS1.2) show the Neanderthal state. These estimates are concordant with human contamination estimates using mtDNA. We concluded that the libraries likely contained small amounts of authentic Neanderthal DNA and decided to perform enrichment capture for mitochondrial sequences.

For those, adapters were trimmed from the two captured libraries using leeHom (36) with the --ancient-dna option, and sequence length was filtered using seqtk with a minimum length threshold of 30 bp (-L 30). Alignment comparisons between the revised Cambridge Reference Sequence (rCRS) and the Altai Neanderthal mitochondrial genome (37) showed that the Starosele sequences aligned more closely with the Altai reference. However, to minimize downstream bias, all subsequent analyses were conducted using the alignment to rCRS.

Reads were aligned to the rCRS using bwa aln, and only those with a mapping quality score greater than 30 were retained. Characteristic ancient DNA damage patterns were confirmed with MapDamage v2.2.2 (38). To further reduce contamination, PMDtools (39) were used to retain only reads with post-mortem damage (PMD) scores equal to or above threshold 1. This filtering step reduced contamination estimates calculated by schmutzi v.1.5.7 (40) to between 0 and 0.5%. Coverage of the merged libraries was visualized using BamPlotter (41).

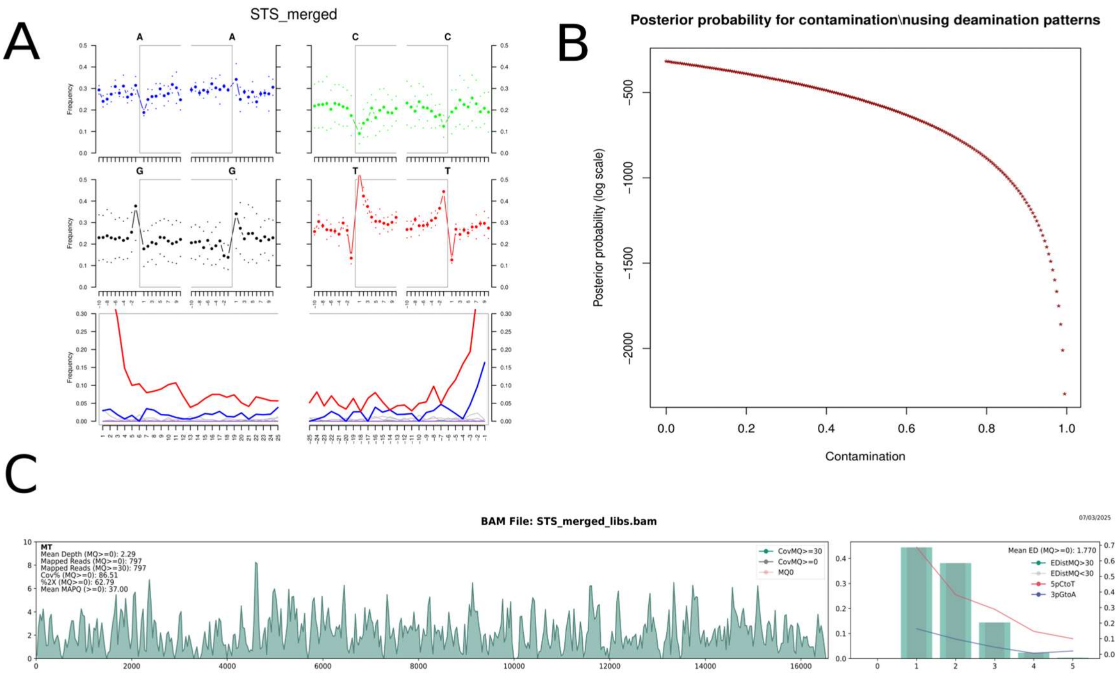

Figure S3. Quality control of Star 1 merged libraries looking at A) pre-PMD filtering deamination pattern, B) post-PMD filtering contamination estimate ranging between 0 and 0.5 with highest probability at 0, and C) aDNA bam plot confirming mean coverage of ~2.29 across 86.51% of the reference genome with mean mapping quality of 37.

A consensus sequence was generated from ~2.28× mitochondrial genome coverage using ANGSD (42) with the following parameters: -doFasta 2 -doCounts 1 -basesPerLine 70 -seed 5.

Multiple sequence alignment (MSA) was performed using MEGA 11 (43) with its in-built MUSCLE algorithm and default parameters. The alignment included Neanderthal and present-day human mitochondrial genomes, with Pan troglodytes as the outgroup. All sequences in the MSA were realigned with rotate (44) to start with the conserved COX1 gene motif ATGTTCGCCGACCGTTGACTATTCTCTACA (45), allowing up to three mismatches.

A phylogenetic tree was constructed using IQ-TREE (46) with 1,000 bootstrap replicates, employing the Kimura 2-parameter model and a gamma distribution to account for rate heterogeneity across sites.

Pairwise nucleotide differences between the Starosele individual and other genomes in the alignment were calculated using a custom Python script, ignoring sites where Starosele had missing data (‘N’) or showed deamination patterns consistent with ancient DNA damage. The script is available on the project’s GitHub repository: https://github.com/KonstantinaChe/Star1_Neanderthal.

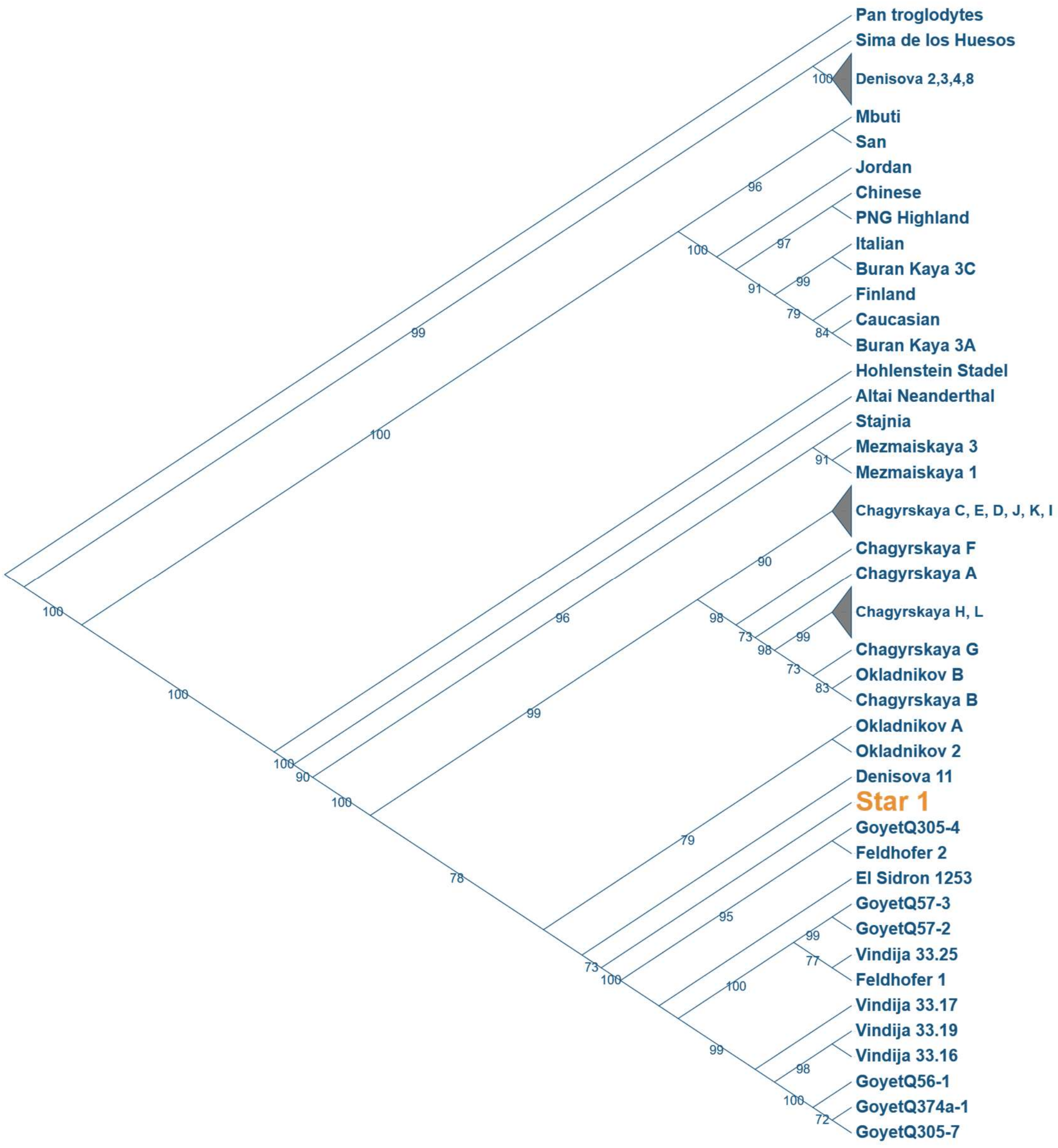

Figure S4. Phylogenetic tree featuring VIE 1203 to other ancient hominins mtDNA data. Bootstrap values are included.

Supplementary information F: Hominins in the broader picture and climate

There are currently many Micoquian sites (some which are still under discussion) known and studied in Eurasia, which can be seen in Fig. S5. Some of the main sites are highlighted within this figure, along with the Neanderthals found in the Altai region, which bears the highest genetic comparison with Star 1. The available current radiometric dates for the Altai

specimens can also be seen in Table S1. The specimens from Okladnikov went under genome capture and radiometric dating methods. Okladnikov 2 was decontaminated before mitochondrial DNA extraction and sequencing was undertaken (39). Radiocarbon dates for layer 3, range from 16,210 BP to 43,300 BP. However, the younger dates are most likely incorrect, due to stratigraphic mixing of content, causing contamination (47, 48). Okladnikov 2, 11 and 15 underwent radiocarbon dating after being treated with a single compound protocol (hydroxyproline) to eliminate any possible contamination (49. 50). Overall, the specimens should be at least 44,000 years old, based on dating at Oxford University (51). Chagyrskaya 6 is a male adult from layer 6b. The specimen was not directly dated but minerals in the sediment from layer 6 were optically dated, to around 52 kyr.

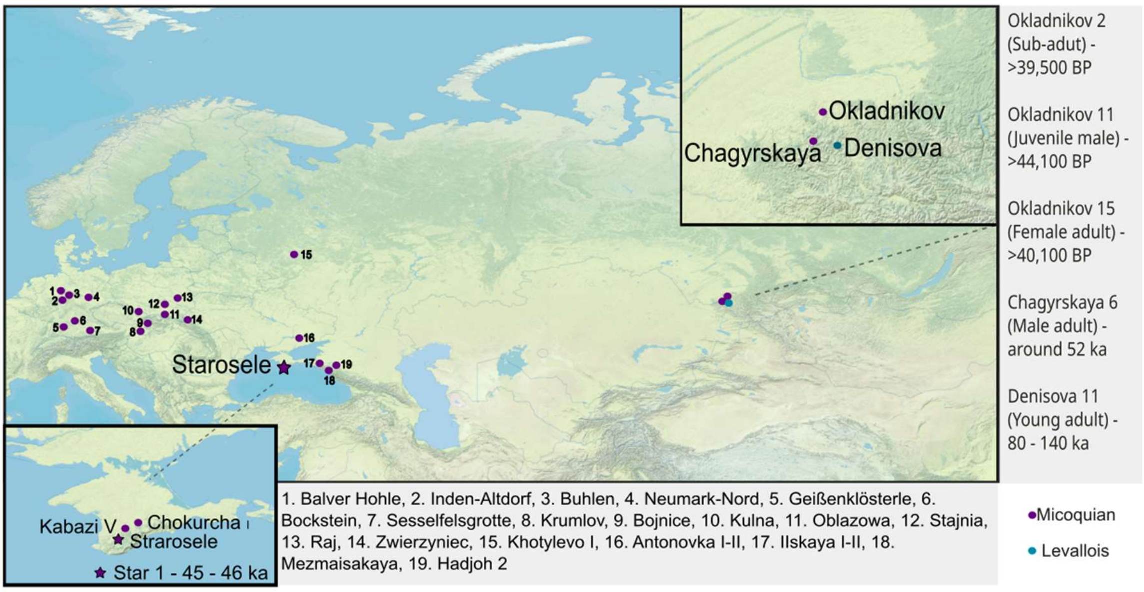

Figure S5. Map showing the location of Starosele, the Altaian sites of Okladnikov, Chagyrskaya and Denisova cluster genetically with Star 1 (more information can be found in Table. S4). Key sites across Eurasia which have Micoquian lithic assemblages are highlighted with purple dots. Map generated with QGIS, and with HCMGIS inputs.

Table S4. Specimens closest (available data at present) related to Star 1 and their radiometric date ranges and methods

**Specimen**	**Age at death**	**Level**	**Dated age**	**Lab code**	**Method**	**Reference**
Starosele	?	1	42,457 cal BP	VIE 1203	Radiocarbon, Ultrafiltration	First published
Okladnikov 2	Sub-adult	3	>39,500 BP	OxA-X 2762-13	Radiocarbon, HYP	Skov et al., 2022 **(51)**
Okladnikov 11	Juvenile Male	2	>44,100 BP	OxA-X 2762-12	Radiocarbon, HYP	Skov et al., 2022 **(51)**
Okladnikov 15	Female adult	2	>40,100 BP	OxA-X 2762-20	Radiocarbon, HYP	Skov et al., 2022 **(51)**
Chagyrskaya 6	Male adult	6b	Around 52 kyr		Optical	Skov et al., 2022 **(51)**
Denisova 11	Teenager/young adult	12	80 to 140 kyr		Genomic data	Douka et al., 2019 **(52)**

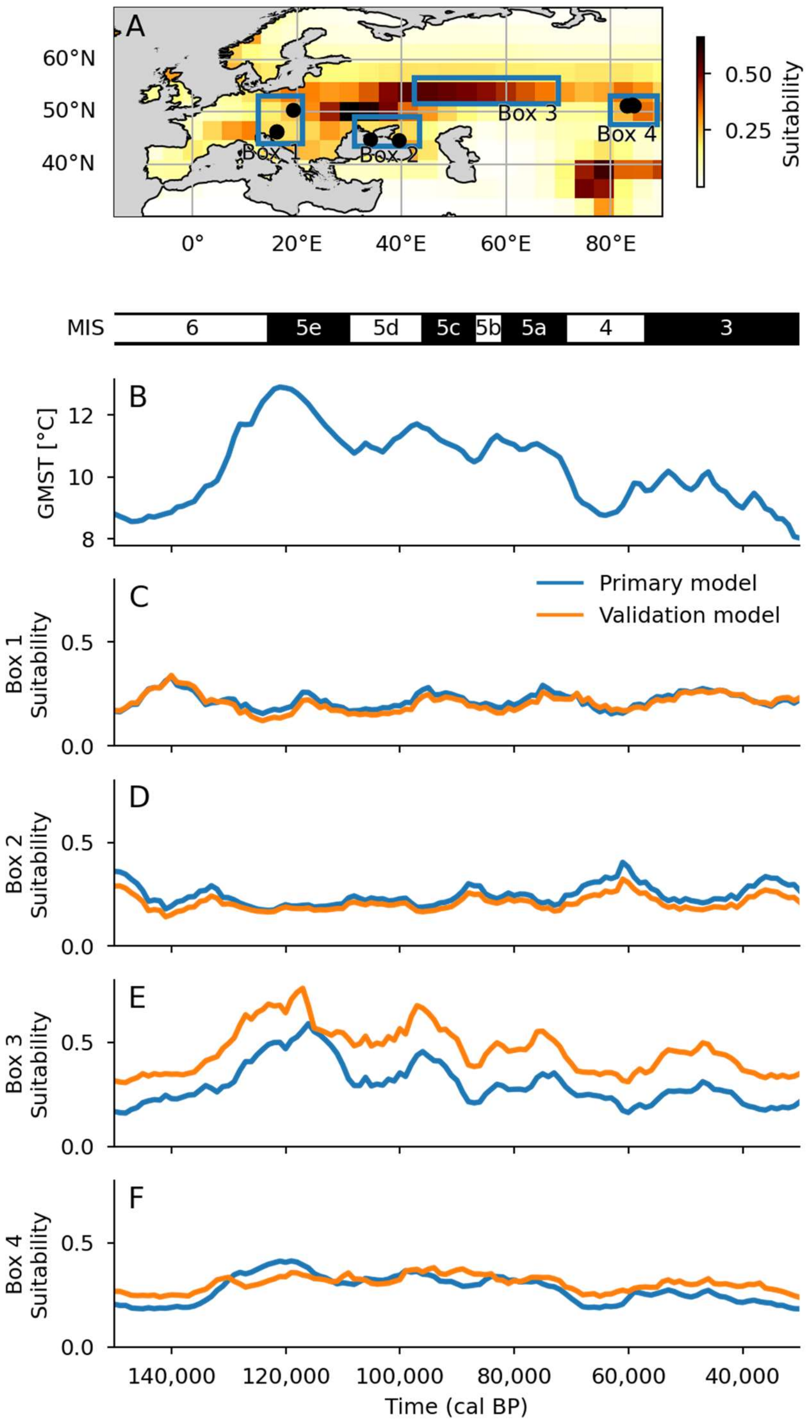

Figure S6. Average habitat suitability validation model (a). With time series of global mean average temperature from transient CESM1.2 3 million years simulation (53, 54) (b), and results of the habitat suitability calculation over the area of Box 1, 2, 3 and 4 (c-f) where the blue line is the average suitability of the primary model and orange line that of the primary model. On top of the timeline the marine isotope stages (MIS) are indicated in black and white, representing warmer and colder periods during the last glacial cycle, respectively.

References in order of citation

1. A Marks, Yu. Demidenko, K. Monigal V. Usik, C. Ferring, “Starosele: the 1993-1996 excavations” in The Middle Palaeolitic of the Western Crimea, vol 1, A. Marks, V. Chabai, Eds. (ERAUL 84, Liège, 1998), pp. 67-99.

2. R. E. M. Hedges, R. A. Housley, P. B. Pettitt, C. Bronk Ramsey, G. J. Van Klinken, Radiocarbon dates from the Oxford AMS system. *Archaeometry* 38, 1, 181-207, (1996). https://doi.org/10.1111/j.1475-4754.1996.tb00770.x
3. E. M. Pigott, T. Uthmeier, V. Chabai, T. F. G. Higham, The Late Middle and Early Upper Palaeolithic in Crimea (Ukraine). Revier of the Neanderthal Refugium Hypothesis. *J. Paleolit. Archaeol.* 7, 27, (2024). https://doi.org/10.1007/s41982-024-00194-y
4. T. Higham, R. M. Jacobi, C. Bronk Ramsey, AMS Radiocarbon Dating of Ancient Bone Using Ultrafiltration. *Radiocarbon*. 48, 2, 179-195 (2016). https://doi.org/10.1017/S0033822200066388
5. B. L. Hardy, M. Kay, A. E. Marks, K. Monigal, Stone tool function at the palaeolithic sites of Starosel'e and Buran Kaya III, Crimea: Behavioural implications. *PNAS*. 98, 19, 10972-10977 (2001). https://doi.org/10.1073/pnas.191384498
6. A. E. Marks, Y. Demidenko, K. Monigal, V. Usik, "Starosele, the 1993–1995 excavations" in The Middle Paleolithic of Western Crimea, vol. 1, A. E. Marks, V. P. Chabai, Eds. (ERAUL 84, Liège,1999), pp. 67–100.
7. A. E. Marks, K. Monigal, V. I. Usik, V, C. R. Ferring, A. Burke, J. Rink, C. McKinney, Starosel'e and the Starosel'e Child: New Excavations, New Results. Curr. *Anthropol*. 38, 1, 112-123 (1997). https://doi.org/10.1086/204591
8. A. E. Marks & V. P Chabai (Eds). The Middle Paleolithic of Western Crimea, Vol 1. Eraul 83, Liège,1998).
9. D. G. Druker, Y. J. Naito, S. Péan, S. Prat, L. Crépin, Y. Chikaraishi, N. Ohkouchi, S. Puaud, M. Lázničková-Galetová, M. Patou-Mathis, A. Yanevich, H. Bocherens, Isotopic analyses suggest mammoth and plaint in the diet of the oldest anatomically modern humans from far southeast Europe. *Sci. Rep.* 7, 6883 (2017). https://doi.org/10.1038/s41598-017-07065-3
10. M. P. Richards, E. Trinkhaus, Isotopic evidence for the diets of European Neanderthals and early modern humans. *Proc. Natl. Acad. Sci.* 106, 38, 16034-16039 (2009). https://doi.org/10.1073/pnas.0903821106
11. K. Jaouen, M. P. Richards, A. L. Cabec, F. Welker, W. Rendau, J.-J. Hublin, M. Soressi, S. Talamo, Exceptionally high δ15N values in collagen single amino acids confirm Neandertals as high-trophic level carnivores. *Proc. Natl. Acad. Sci. U.S.A.* 116, 11, 4928-4933 (2019). https://doi.org/10.1073/pnas.1814087116
12. M. Patou-Mathis, "Analyse Archéozoologique de l'Unite II, Niveaux II/7AB à IIA/4B" in Kabazi II: the 70000 years since the Last Interglacial. Palaeolithic site of Crimea, Vol. 2, V. Chabai, J. Richter, T. Uthmeier, Eds. (Simferopol–Cologne, Shlyakh, 2006), pp. 37-62.
13. A. Burke, The View from Starosel'e: Faunal Exploitation at a Middle Palaeolithic Site in Western Crimea. *Int. J. Osteoarchaeol.* 10, 325-355 (2000).
14. J. D. Speth, E. Morin, Putrid Meat in the Tropics: It Wasn't Just for Inuit. *PaleoAnthropology.* 2, 327-383. https://doi.org/10.48738/2022.iss2.114
15. S. Brown, N. Wang, A. Oertle, M. B. Kozlikin, M. V. Shunkov, A. P. Derevianko, D. Comeskey, B. Jope-Street, V. L. Harvey, M. P. Chowdhury, M. Buckley, T. Higham, K. Douka, Zooarchaeology through the lends of collagen fingerprinting at Denisova Cave. *Sci. Rep*. 11, 15457 (2021). https://doi.org/10.1038/s41598-021-94731-2
16. I. C. C. Von Holstein, S, P. Ashby, N. L. Van Doorn, S. M. Sachs, M. Meirai, I. Barnes, M. Buckley, M. Meiri, I. Barnes, A. Brundle, M. J. Collins, Searching for Scandinavians in pre-Viking Scotland: molecular fingerprinting of Early Medieval combs. *J. Archaeol. Sci*. 41, 1–6 (2014). https://doi.org/10.1016/j.jas.2013.07.026
17. C. Gilbert, V. Krupicka, F. Galluzzi, A. Popowich, K. Bathany, S. Claverol, J. Arslanoglu, C. Tokarski, Species identification of ivory and bone museum objects using minimally invasive proteomics. *Sci. Adv*. 10, eadi9028 (2024). https://doi.org/10.1126/sciadv.adi9028
18. Y. Kumazawa, T. Taga, K. Iwai, Y.I Koyama, A Rapid and Simple LC-MS Method Using Collagen Marker Peptides for Identification of the Animal Source of Leather. J Agric *Food chem*. 64, 30, 6051-6057 (2019). https://doi.org/10.1021/acs.jafc.6b02132
19. S. Fiddyment, B. Holsinger, C. Ruzzier, A. Devine, A. Binois, U. Albarella, R. Fischer, E. Nichols, A. Curtis, E. Cheese, M. D. Teasdale, C. Checkley-Scott, S. J. Milner, K. M. Rudy, E. J. Johnson, J. Vnoućek, M. Garrison, S. McGrory, D. G. Bradley, M. J.

Collins, Animal origin of 13th-century uterine vellum revealed using noninvasive peptide fingerprinting. *PNAS*. 112, 15066–15071 (2015). https://doi.org/10.1073/pnas.1512264112
20. J. Sakalauskaite, F. Marin, B. Pergolizzi, B. Demarchi, Shell palaeoproteomics: First application of peptide mass fingerprinting for the rapid identification of mollusc shells in archaeology. *J. Proteom*. 227, 103920 (2020). https://doi.org/10.1016/j.jprot.2020.103920
21. S. Brown, T. Higham, V. Slon, S. Pääbo, M. Meyer, K. Douka, F. Brock, D. Comeskey, N. Procopio, M. Shunkov, A. Derevianko, M. Buckley, Identification of a new hominin bone from Denisova Cave, Siberia using collagen fingerprinting and mitochondrial DNA analysis. *Sci. Rep*. 6, 23559 (2016). https://doi.org/10.1038/srep23559
22. A. N. Coutu, G. Whitelaw, P. L. Roux, J. Sealy, (2016). Earliest Evidence for the Ivory Trade in Southern Africa: Isotopic and ZooMS analysis of Seventh-Tenth Century AD Ivory from KwaZulu-natal. *Afr. Res. Rev*. 33, 4, 411-435 (2016). https://doi.org/10.1007/s10437-016-9232-0
23. N. L. Martisius, F. Welker, T. Dogandžić, M. N. Grote, W. Rendu, V. Sinet-Mathiot, A. Wilcke, A, S. J. P. McPherron, M. Soressi, T. E. Steele, Non-destructive ZooMS identification reveals strategic bone tool raw material selection by Neandertals. *Sci. Rep*. 10, 7746 (2020). https://doi.org/10.1038/s41598-020-64358-w
24. K. McGrath, K. Rowsell, C. G. St-Pierre, A. Tedder, G. Foody, C. Roberts, C. Speller, M. Collins, Identifying Archaeological Bone via Non-Destructive ZooMS and the Materiality of Symbolic Expression: Examples from Iroquoian Bone Points. *Sci. Rep*. 9, 11027 (2019) https://doi.org/10.1038/s41598-019-47299-x
25. J. Fellow Yates, It Will Not Be Possible To Use Zooarchaeology By Mass Spectrometry (ZooMS) To Identify Species In Samples Of Cremated Bone That Have Been Burnt Higher Than 155oC. University of York, York, thesis (2013). https://doi.org10.13140/RG.2.1.3370.7043
26. E. Végh, K Douka, SpecieScan: semi-automated taxonomic identification of bone collagen peptides from MALDI-TF-MS. *Bioinformatics*. 4, 40, 3, btae054 (2024). https://doi.org/10.1093/bioinformatics/btae054.
27. K. Pawłowska, MIS 3–1 fauna from Krosinko: Implications for the past biogeography,chronology and palaeoenvironments of Poland. *Quart. Int*. 632, 79-93, (2022). https://doi.org/10.1016/j.quaint.2022.02.006.
28. M. A. Julien, H. Bocherens, A. Burke, D. G. Drucker, M. Patou-mathis, O. Krotova, S. Péan, Were European steppe bison migratory? 18O, 13C and Sr intra-tooth isotopic variations applied to a palaeoethological reconstruction. *Quat. Int.* 271, 106-119, (2012). https://doi.org/10.1016/j.quaint.2012.06.011.
29. M. Gąsiorowski, H. Hercman, B. Ridush, K. Stefaniak, Environment and climate of the Crimean Mountains during the Late Pleistocene inferred from stable isotope analysis of red deer (Cervus elaphus) bones from the Emine-Bair-Khosar Cave. *Quat. Int*. Vol 326-327, 243-249, (2014). https://doi.org/10.1016/j.quaint.2013.12.020.
30. J. Dabney, M. Knapp, I. Glocke, M.-T. Gansauge, A. Weihmann, B. Nickel, C. Valdiosera, N. García, S. Pääbo, J.-L. Arsuaga, M. Meyer, Complete mitochondrial genome sequence of a Middle Pleistocene cave bear reconstructed from ultrashort DNA fragments. *Proc. Natl. Acad. Sci. U.S.A.* 110, 39, 15758-15763 (2013). https://doi.org/10.1073/pnas.1314445110.
31. P. Korlević, T. Gerber, M.-T. Gansauge, M. Hajdinjak, S. Nagel, A. Aximu-Petri, M. Meyer, Reducing Microbial and Human Contamination in DNA Extractions from Ancient Bones and Teeth. *Biotechniques*. 59, 2, 57-93 (2015). https://doi.org/10.2144/000114320
32. J. D. Kapp, R. E. Green, B. Shapiro, A Fast and Efficient Single-stranded Genomic Library Preparation Method Optimized for Ancient DNA. *J. Heredity*. 112, 3, 241-249 (2021). https://doi.org/10.1093/jhered/esab012
33. L. F. K. Kuderna, H. Gao, M. C. Janiak, M. Kuhlwilm, J. D. Orkin, T. Bataillon, S. Manu, A. Valenzuela, J. Bergman, M. Rousselle, F. E. Silva, L. Agueda, J. Blanc, M Gut, D. De Vries, I. Goodhead, R. A. Harris, M. Raveendran, A. Jensen, I. S. Chuma, J. E. Horvath, C. Hvilsom, D. Juan, P. Frandsen, J. G. Schraiber, F. R. de Melo, F. Bertuol, H. Byrne, I. Sampaio, I. Farias, J. Valsecchi, M. Messias, M. N. F. da Silva, M. Trivedi, R. Rossi, T. Hrbek, N. Andriaholinirina, C. J. Rabarivola, A.

Zaramody, C. J. Jolly, J. Phillips-Conroy, G. Wilkerson, C. Abee, J. H. Simmons, E. Fernandez-Duque, S. Kanthaswamy, F. Shiferaw, D. Wu, L. Zhou, Y. Shao, G. Zhang, J. D. Keyyu, S. Knauf, M. D. Le, E. Lizano, S. Merker, A. Navarro, T. Nadler, C. C. Khor, J. Lee, P. Tan, W. K. Lim, A. C. Kitchener, D. Zinner, I. Gut, A. D. Melin, K. Guschanski, M. H. Schierup, R. M. D. Beck, G. Umapathy, C. Roos, J. P. Boubli, J. Rogers, K. Kai-How. Farh, T. M. Bonet, A global catalog of whole-genome diversity from 233 primate species. *Science.* 380, 6648, 906–913 (2023). https://doi.org/10.1126/science.abn7829

34. P. Danecek, J. Bonfield, J. Liddle, J. Marshall, V. Ohan, M. O. Pollard, A. Whitwham, T. Keane, S. A. McCrthy, R. M. Davies, H. Li, Twelve years of SAMtools and BCFtools. *Gigascience*. 10, 2 (2021). https://doi.org/10.1093/gigascience/giab008
35. M. Kuhlwilm, C. Fontsere, S. Han, M. Alvarez-Estape, T. Marques-Bonet, HuConTest: Testing Human Contamination in Great Ape samples. *Geonome. Biol. Evol*. 13, 6 (2021). https://doi.org/10.1093/gbe/evab117
36. G. Renaud, U. Stenzel, J. Kelso, leeHom: adaptor trimming and merging for Illumina sequencing reads. *Nucleic Acids Res*. 42, 18, e141 (2014). https://doi.org/10.1093/nar/gku699
37. K. Prüfer, F. Racimo, N. Patterson, F. Jay, S. Sankararaman, S. Sawyer, A. Heinze, G. Renaud, P. H. Sudmant, C. De Filippo, H. Li, S. Mallick, M. Dannemann, Q. Fu, M. Kircher, M. Kuhlwilm, M. Lachmann, M. Meyer, M. Ongyerth, M. Siebauer, C. Theunert, A. Tandon, P. Moorjani, J. Pickrell, J. C. Mullikin, S. H. Vohr, R. E. Green, I. Hellmann, P. L. F. Johnson, H. Blanche, H. Cann, J. O. Kitzman, J. Shendure, E. E. Eichler, E. S. Lein, T. E. Bakken, L. V. Golovanova, V. B. Doronichev, M. V. Shunkov, A. P. Derevianko, B. Viola, M. Slatkin, D. Reich 19, J. Kelso, S. Pääbo, The complete genome sequence of a Neanderthal from the Altai Mountains. *Nature*. 505, 7481, 43–49 (2013). https://doi.org/10.1038/nature12886
38. H. Jónsson, A. Ginolhac, M. Schubert, P. L. F. Johnson, L. Orlando, mapDamage2.0: fast approximate Bayesian estimates of ancient DNA damage parameters. *Bioinformatics*. 29, 13, 1682–1684 (2013).
39. P. Skoglund, B. H. Northoff, M. V. Shunkov, A. P. Derevianko, S. Pääbo, J. Krause, M. Jakobsson, Separating endogenous ancient DNA from modern day contamination in a Siberian Neandertal. *Proc. Natl. Acad. Sci*. 111, 6, 2229–2234 (2014). https://doi.org/10.1073/pnas.1318934111
40. G. Renaud, V. Slon, A. T. Duggan, J. Kelso, Schmutzi: estimation of contamination and endogenous mitochondrial consensus calling for ancient DNA. *Genome Biol*. 16, 1, (2015). https://doi.org/10.1186/s13059-015-0776-0
41. M. Guellil, MeriamGuellil/aDNA-BAMPlotter: aDNA-BAMPlotter. Zenodo (2021). https://doi.org/10.5281/zenodo.5676092
42. T. S. Korneliussen, A. Albrechtsen, R. Nielsen, ANGSD: Analysis of Next Generation Sequencing Data*. BMC Bioinformatics*. 15, 1 (2014). https://doi.org/10.1186/s12859-014-0356-4
43. K. Tamura, G. Stecher, S. Kumar, MEGA11: Molecular Evolutionary Genetics Analysis Version 11. *Mol. Biol. Evol*. 38, 7, 3022–3027 (2021). https://doi.org/10.1093/molbev/msab120
44. R. Durbin, B. De Sanctis, M. Blumer, Rotate: A command-line program to rotate circular DNA sequences to start at a given position or string. *Wellcome Open Research*, 8, 401 (2023). https://doi.org/10.12688/wellcomeopenres.19568.1
45. P. D. Hebert, S. Ratnasingham, J. R. De Waard, Barcoding animal life: cytochrome c oxidase subunit 1 divergences among closely related species. *Proc. R. Soc. B. Biol. Sci*. 270, (suppl_1) (2023). https://doi.org/10.1098/rsbl.2003.0025
46. J. Trifinopoulos, L. Nguyen, A. Von Haeseler, B. Q. Minh, W-IQ-TREE: a fast online phylogenetic tool for maximum likelihood analysis. *Nucleic. Acids. Res*. 44, W1 W232–W235 (2016). https://doi.org/10.1093/nar/gkw256
47. S. A. Vasil’ev, Y. V. Kuzmin, L. A. Orlova, V N. Dementiev, Radiocarbon-based chronology of the Palaeolithic in Siberia and its relevance to the peopling of the world. *Radiocarbon*. 44, 2, 503-530, (2022). https://doi.org/10.1017/S0033822200031878.
48. J. Krause, L. Orlando, D. Serre, B. Viola, K. Prüfer, M. P. Richards, J.-J. Hublin, C. Hänni, A. P. Derevianko, S. Pääbo, Neanderthals in Central Asia and Siberia. *Nature*. 448, 18, 902-904, (2007). Doi: https://doi.org/10.1038/nature06193

49. T. Devièse, D. Comeskey, J. McCullagh, C. Bronk Ramsey, T. Higham, New protocol for compound-specific radiocarbon analysis of archaeological bones. *RCM*. 32, 373-379, (2017). https://doi.org/10.1002/rcm.8047
50. A. Marom, J. S. O. McCullagh, T.F. G. Higham, A. A. Sinitsyn, R. E. M. Hedges, Single amino acid radiocarbon dating of Upper Palaeolithic modern humans. *PNAS*. 109, 18, 6787-6881, (2012). https://doi.org/10.1073/pnas.11163 28109
51. L. Skov, S. Peyrégne, D. Popli, L. N. M. Lasi, T. Devièse, V. Slon, E. I. Zavala, M. Hajdinjak, A. P. Sümer, S. Grote, A. B. Mesa, D. L. Herráez, B. Nickel, S. Nagel, J. Richteer, E. Essel, M. Gansauge, A. Schmidt, P. Korlevic, D. Comeskey, A. P. Derevianko, A. Kharevich, S. V. Markin, S. Talemo, K. Douka, M. T. Krajcarz, R. G. Roberts. T. Higham, B. Viola, A. I. Krivoshapkin, K. A. Kolobova, J. Kelso, M. Meyer, S. Pääbo, B. M. Peter, Genetic insights into the social organization of Neanderthals. *Nature*. 610, 519-525 (2022). https://doi.org/10.1038/s41586-022-05283-y.
52. K. Douka, V. Slon, Z. Jacobs, C. Bronk Ramsey, M. V. Shunkov, A. P. Derevianko, F. Mafessoni, M. B. Kozlikin, B. Li, R. Grün, D. Comeskey, T. Devièse, S. Brown, B. Viola, L. Kinsley, M. Buckley, M. Meyer, R. G. Roberts, S. Pääbo, J. Kelso, T. Higham, Age estimates for hominin fossils and the onset of the upper Palaeolithic at Denisova Cave. *Nature*. 565, 640-644, (2019). https://doi.org/10.1038/s41586-018-0870-z
